# Supplementary material for: Field Evaluation of a Wearable Instrumented Headband Designed for Measuring Head Kinematics
Source: Ann Biomed Eng. 2026 Mar 13;54(10):3622–37. doi: 10.1007/s10439-026-04051-7 (PMC13649856; doi:10.1007/s10439-026-04051-7)
Supplement: Supplementary file 1 [file 10439_2026_4051_MOESM1_ESM.pdf]

Supplementary Materials:
Field evaluation of a wearable instrumented headband designed for measuring head kinematics

Journal: Annals of Biomedical Engineering

Anu Tripathi[1], Yang Wan[2], Zhu Zhiren[3], Furkan Camci[4], Sheila Turcsanyi[4], Jeneel Pravin Kachhadiya[5], Mauricio Araiza Canizales[6], Alison Brooks[7], Haneesh Kesari[2], Joseph Andrews[6], Traci Snedden[8], Peter Ferrazzano[4], Christian Franck[6], Rika Wright Carlsen[1]*

[1]Department of Engineering, Robert Morris University, Moon Township, PA, USA.
[2]School of Engineering, Brown University, Providence, RI, USA.
[3]Department of Mechanical Engineering, University of Michigan, Ann Arbor, MI, USA.
[4]Waisman Center, University of Wisconsin–Madison, Madison, WI, USA.
[5]Department of Electrical and Computer Engineering, University of Wisconsin–Madison, Madison, WI, USA.
[6]Department of Mechanical Engineering, University of Wisconsin–Madison, Madison, WI, USA.
[7]Department of Orthopedics and Rehabilitation, University of Wisconsin–Madison, Madison, WI, USA.
[8]College of Nursing, University of Colorado Anschutz Medical Campus, Aurora, CO, USA

*Corresponding author: carlsen@rmu.edu;
Contributing authors: tripathia@rmu.edu; y.wan@northeastern.edu; zhiren@umich.edu; camci@wustl.edu; saturcsanyi@wisc.edu; jeneel.kachhadiya@wisc.edu; araizacaniza@wisc.edu;brooks@ortho.wisc.edu; haneesh_kesari@brown.edu; joseph.andrews@wisc.edu; traci.snedden@cuanschutz.edu; ferrazzano@pediatrics.wisc.edu; cfranck@wisc.edu;

1. CHAMP Checklist

Checklist item	Explanation Example(s)	Reported on Page No.
1. Sensor Technology and Specifications		
(1a) Device model name		Page 3, Section 2.1
(1b) Sensor type		Page 3, Section 2.1
(1c) Sensor sample rate		Page 3, Section 2.1
1d) Sensor magnitude Range		Page 3, Section 2.1
(1e) If applicable, device hardware/ firmware version number		Page 3, Section 2.1
(1f) Recording trigger Threshold	Not applicable; Data recorded continuously<br><br>“The beginning of the head impact is defined as the time when the head linear acceleration exceeds 3 g for 3 ms”	N/A<br><br>Page 6; second paragraph

(1g) Pre- and post-trigger Duration	Data recorded continuously<br><br>“We report data from 50 ms before and 150 ms after the impact”	N/A
(1h) Device form factor and attachment		Page 3, Section 2.1
(1i) If applicable, device fitting procedures		Page 4, Section 2.3
(1j) If applicable, helmet manufacturer/ model name		N/A
(1k) Evidence of device kinematic validation	“The headband performance was evaluated against the reference DTS sensors affixed to the ATD in the laboratory settings in a previous study [19].”	Page 2, Introduction para 2
2. On-Field Logistics		
(2a) Participants	“adult female, former Division I player”	Page 4, Section 2.3
(2b) Study settings/location		Page 4, Section 2.3
2c) Data collection Dates		Page 4, Section 2.3

(2d) Device usage		Page 4, Section 2.3
3. Head Acceleration Event Verification		
(3a) Method of impact verification		Page 4, Section 2.3
(3b) Time synchronization method		Page 4, section 2.3
Data Windowing		
(4a) Temporal windowing		Page 4, Section 2.3
(4b) Temporospatial windowing		N/A
(4c) Kinematic windowing		N/A
5. Video Verification		
(5a) Type of video review, if applicable	Manual	Page 4, Section 2.3
(5b) Video recording parameters, if applicable	30 fps	Page 4, Section 2.3

(5c) Video/observer Review parameters, if applicable	“Videos were reviewed during analysis to ensure header type correlation with the sensors data”.	Page 4, Section 2.3
(5d) Contextual information of head impacts	“The athlete went through a series of typical soccer headers: throw-ins, goal kicks, and corner kicks”	Page 4, Section 2.3
6. Advanced Post-processing		
(6a) Data transformation		Pages 4 – 7; Section 2.4
(6b) Kinematic data filtering	The adaptive filtering method	Page 5-6, Section 2.4.1 Appendix A
(6c) Other post-processing techniques	A3G1 algorithm	Page 6-7; Section 2.4.2
7. Data Reporting		
(7a) Event definition	“The beginning of the head impact is defined as the time when the head linear acceleration exceeds 3 g for 3ms”	Page 6; second paragraph

(7b) Device-recorded Events	The kinematics data for all 18 impacts were recorded on both the mouthpiece and the headband sensors	Page 9; Section 3
(7c) Head acceleration Events	Same as 7b	Page 9; Section 3
(7d) Device performance	Data was recorded continuously on the headband sensors.<br>So, false negative can't be computed.	N/A
(7e) Athlete exposures	"The athlete fitted with the instrumented headband went through a series of six controlled<br>throw-ins, goal-kicks, and corner-kicks."	Page 9; Section 3, first paragraph
(7f) Outcomes measures, including equations used to derive outcomes, if applicable		Page 6;<br>Section 2.4 (first para)
(7g) If relevant, equations used to derive outcomes		N/A
(7h) If relevant, categories of direction of impact		N/A

2. End time sensitivity analysis

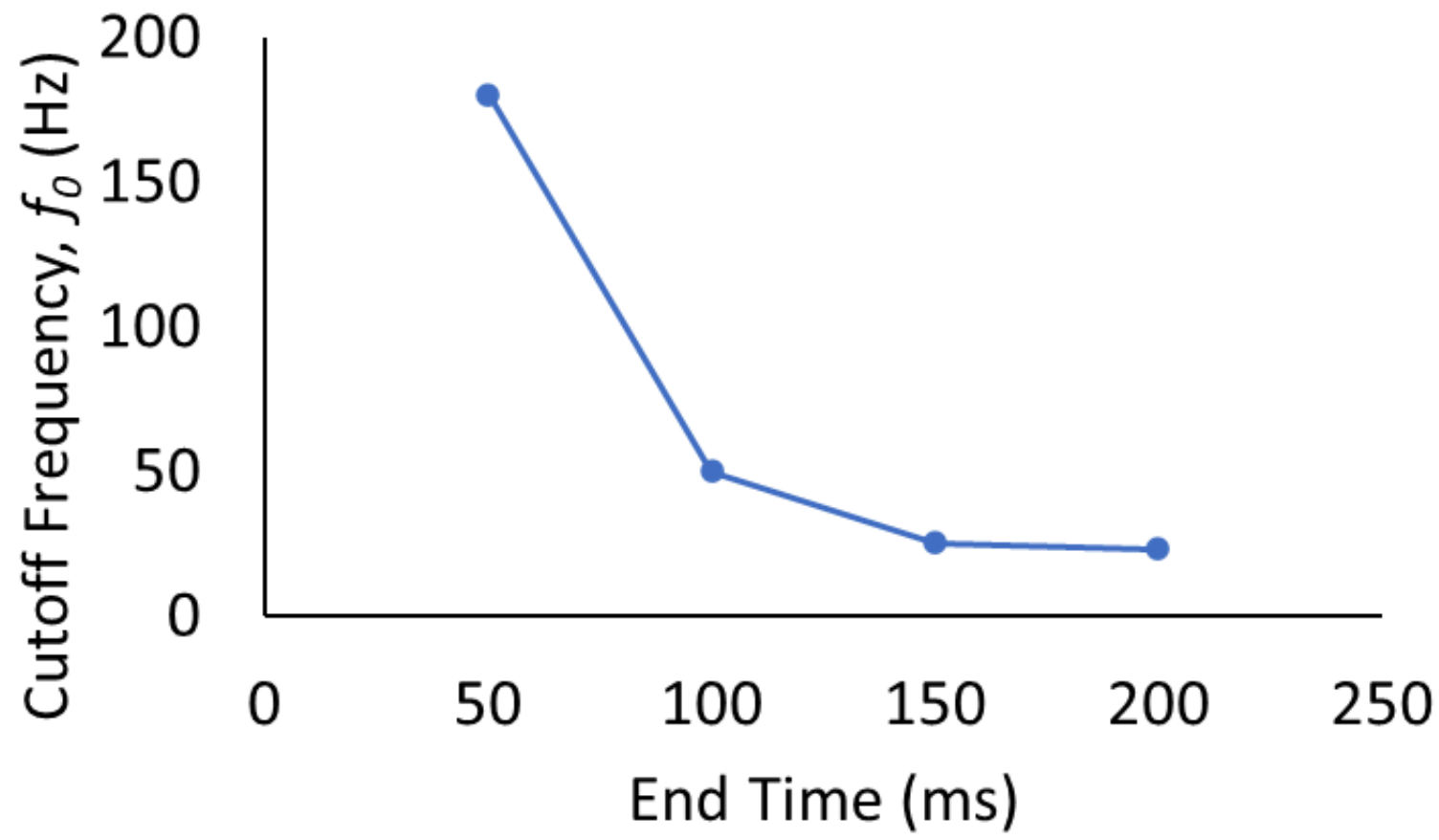

Figure S1: Sensitivity analysis of cutoff frequency, f_0, as a function of end time used in the adaptive filtering algorithm for a representative goal-kick header (sagittal component). The cutoff-frequency converges as the time increases, and therefore, 150 ms was selected as the end time for the analysis.

3. All Data: Angular Velocity Time Histories

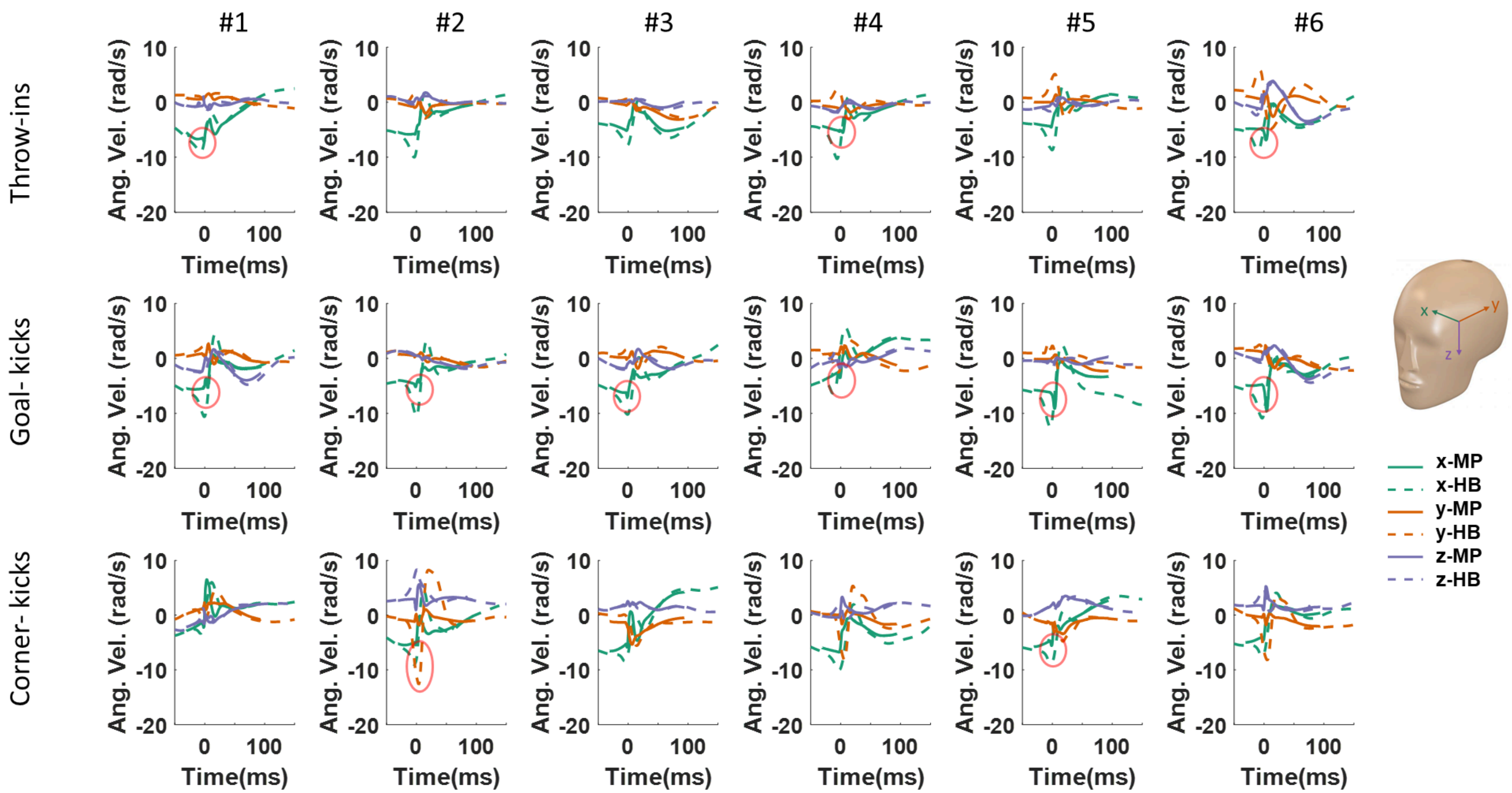

Figure S2: Time history curves of angular velocity reconstruction from the mouthpiece (MP) (as per Rich et al. [16]) and the headband (HB) reconstruction (Section 2.4.1) for all 18 headers. The red circles indicate short-duration pulse features in the mouthpiece data at the beginning of the impact (Section 4.1).

4. All Data: Angular Acceleration Time Histories (Differentiation)

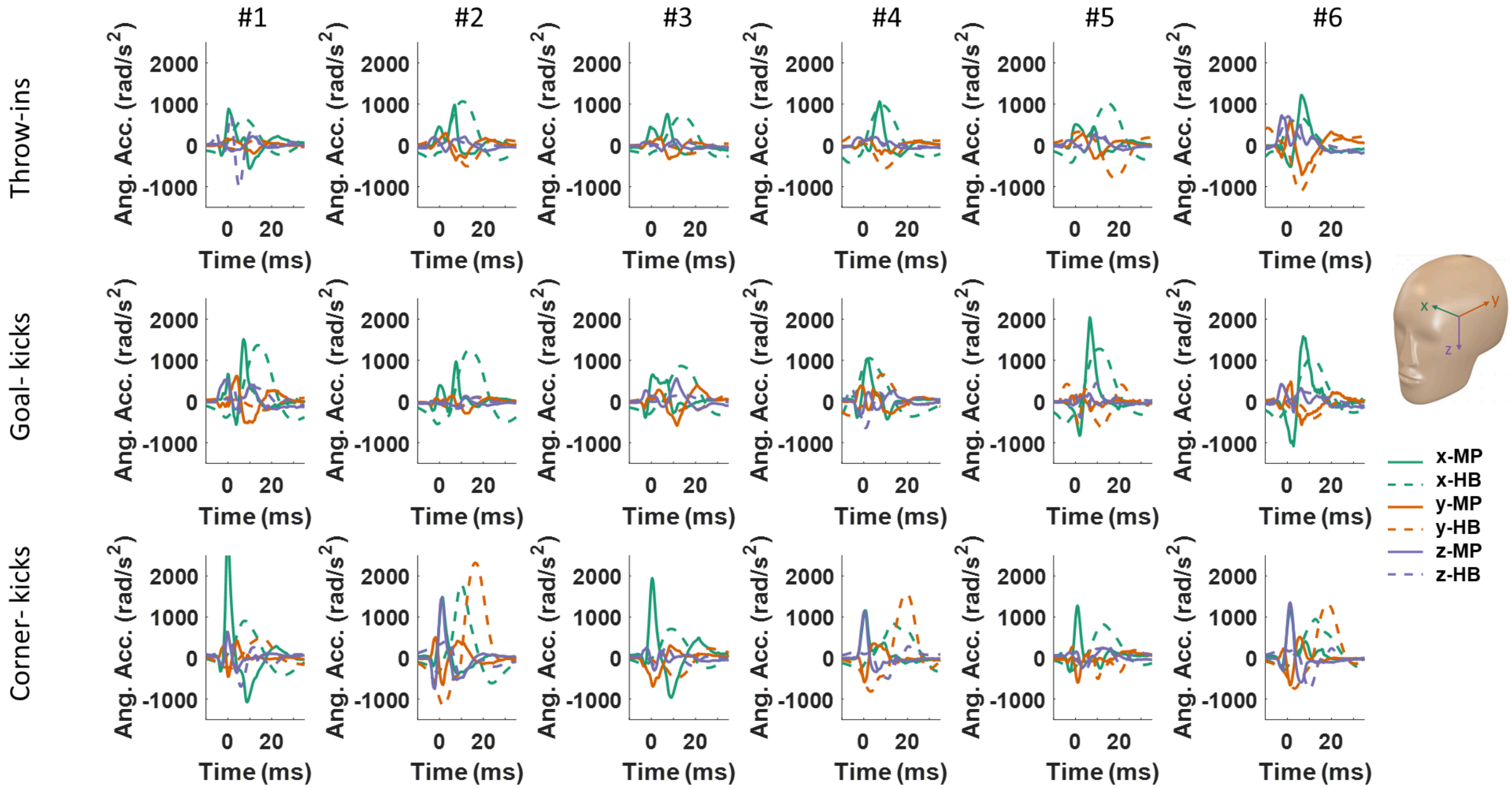

Figure S3: Time history curves of angular accelerations from the mouthpiece (MP) and the headband (HB) reconstruction for all 18 headers, with both devices using the differentiation method (Section 2.4.2).

5. All Data: Angular Acceleration (A3G1)

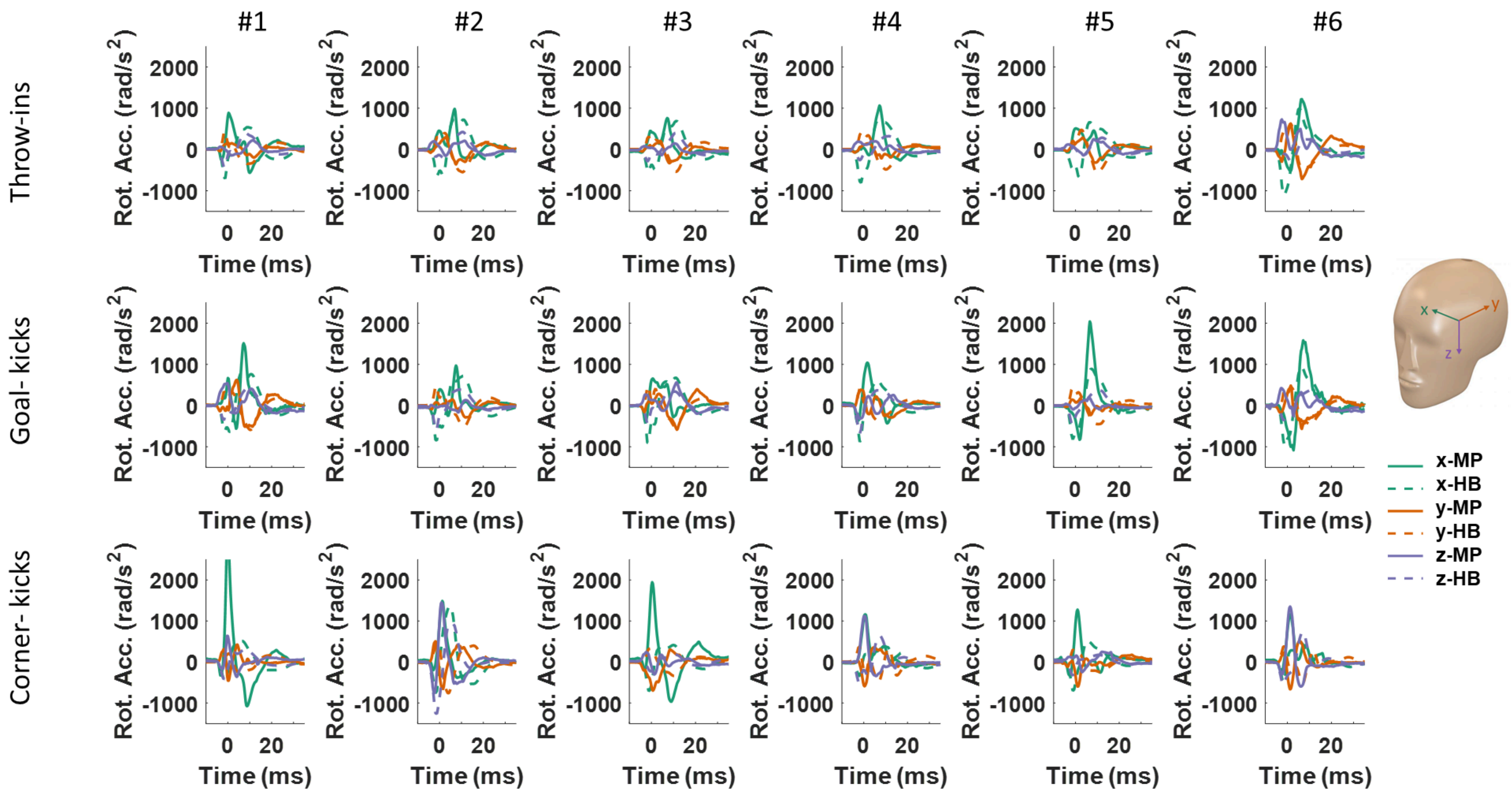

Figure S4: Time history curves of angular accelerations from the mouthpiece (MP) (using differentiation method [16]) and the headband (HB) reconstruction using the A3G1 method for all 18 headers (Section 2.4.2).

6. **All Data: Translational Acceleration**

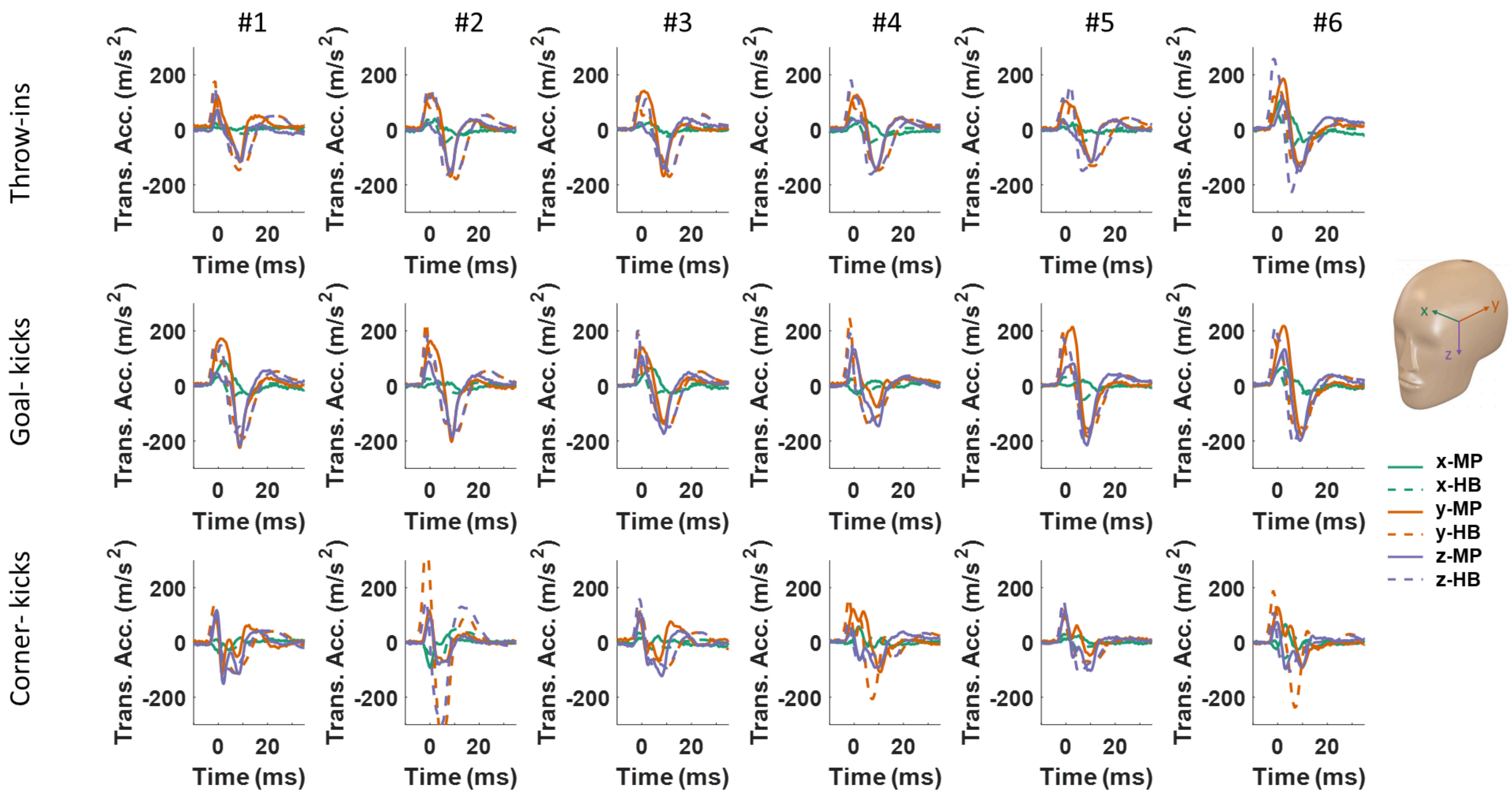

Figure S5: Time history curves of translational accelerations (at the location of the mouthpiece) from the mouthpiece (MP) (as measured by the mouthpiece and filtered as per Rich et al. [16]) and the headband (HB) reconstruction using the A3G1 method for all 18 headers (Section 2.4.2).

7. Effect of Slipping on Sensor Kinematics Profile

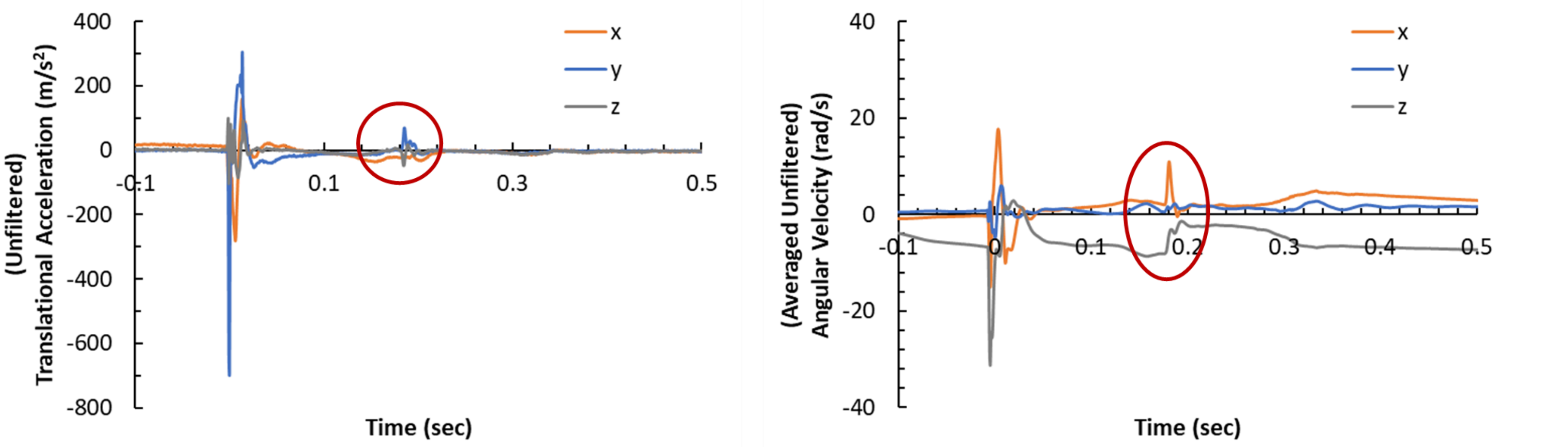

Figure S6: Out of the 18 headers performed, headband slipping was observed in one case (goal kick #5), where the headband slipped and dropped to the ground. The slipping event did not affect the initial part of the soccer ball impact signal used in the analysis, which is not visibly different from other impacts (Figure S2-S5). The sensor data shows high peaks 200 ms after the initial impact, which corresponds to the slipping event (circled).

8. Effect of Acceleration Time History on Brain Strains

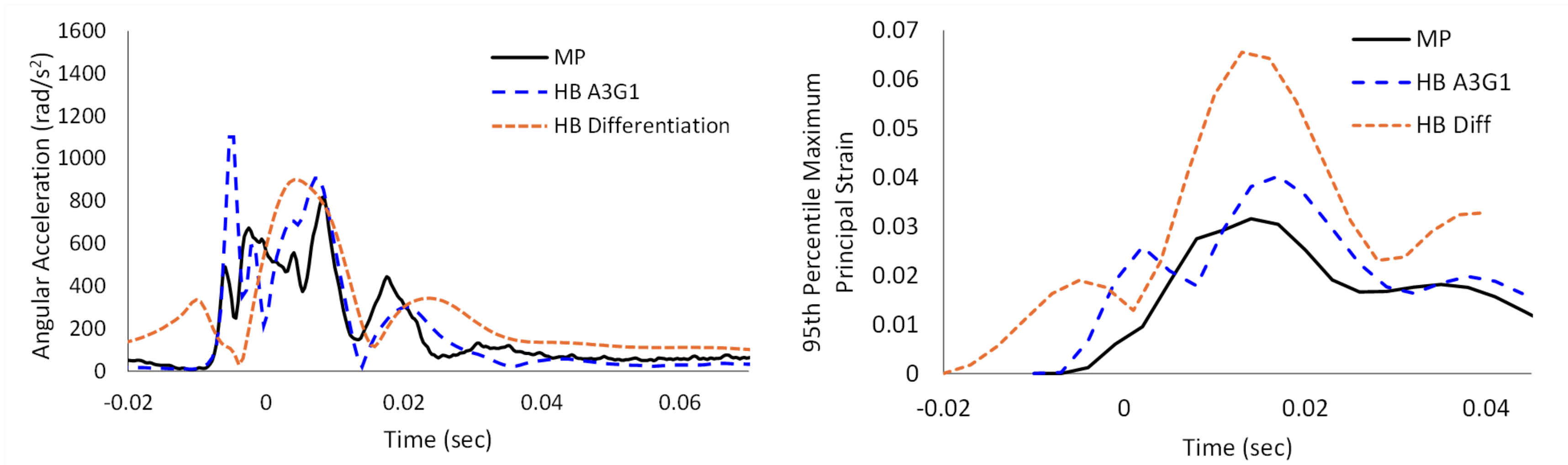

(a) (b)

Figure S7: Three finite element (FE) simulations were performed based on the angular accelerations obtained from the headband differentiation reconstruction, headband A3G1 reconstruction, and mouthpiece differentiation reconstruction. a) Angular accelerations for the three cases are shown. b) The brain strains (95th percentile maximum principal strain, MPS95) from the three FE simulations are shown. These results show that the error in the peak angular accelerations from the differentiation and A3G1 methods is 10% and 34.5%, respectively, relative to the mouthguard data, while the error in the brain strain is 100% for the headband differentiation reconstruction and 27% for the A3G1 reconstruction relative to the mouthguard results.
